# Comment on “Evaluation of Subfoveal Choroidal Thickness in Internal Carotid Artery Stenosis”

**DOI:** 10.1155/2016/7575643

**Published:** 2016-09-01

**Authors:** Salih Uzun, Yakup Yesilkaya, Emre Pehlivan

**Affiliations:** ^1^Department of Ophthalmology, Etimesgut Military Hospital, 06790 Ankara, Turkey; ^2^Department of Radiology, Etimesgut Military Hospital, 06790 Ankara, Turkey; ^3^Department of Ophthalmology, Eskisehir Military Hospital, Eskisehir, Turkey

We have read the article entitled “Evaluation of Subfoveal Choroidal Thickness in Internal Carotid Artery Stenosis” by Akçay et al. with great interest [1]. The authors researched the relationship between internal carotid artery (ICA) stenosis and subfoveal choroidal thickness (CT) in the elderly population. Forty-two eyes of 21 patients with more than 70% ICA stenosis (Group 1) and less than 70% stenosis (Group 2) were recruited for this study. They discovered that the mean subfoveal CT was 231.9 ± 44.6 *μ*m in Group 1 and 216.2 ± 46.8 *μ*m in Group 2, which was significantly thinner. In addition, statistically significant positive correlation was found between the percentage of internal carotid artery stenosis and subfoveal CT. We congratulate the authors for their valuable study and we would like to ask for more details and contributions to the article.

Primarily, various local and systemic physiologic/pathologic conditions and environmental factors may have effect on CT. It has been shown in the literature that age, sex, systemic/local diseases and their treatment, drug use, intraocular pressure, refractive error, and many other factors have effect on CT [2, 3]. Although Esen et al. have specified many factors in their study, we would like to ask about some points which may be considered important to the authors.

Firstly, before OCT measurements we consider that the axial length of the globe, sleeping and exercising habits of the patients, and their inclination to consume alcohol or drinks with/without caffeine must be known. We also wonder if body mass indexes were considered for parameters as these can considerably change CT status [2, 3].

Secondly, significant diurnal variation is shown in CT, and we have realized that Akçay et al. have not mentioned diurnal variation in their articles. The thickness of choroid is able to rise by 50% in an hour and increase by four times in few days [2]. It has been demonstrated by Kee et al. that the choroid can get thinner very rapidly, by almost 100 *μ*m in 3-4 h in chicks [4]. Subfoveal CT in healthy subjects has been researched by Usui et al. and they evaluated subfoveal CT every 3 hours over a period of 24 hours. It has been found by them that average subfoveal CT was thinnest (271.9 ± 103.5 *μ*m) at 6 PM and thickest (290.8 ± 110.8 *μ*m) at 3 AM. It has been indicated by Usui et al. in that study that CT's diurnal variation is able to be up to 65 *μ*m (range: 8–65 *μ*m), and subfoveal CT in all eyes had a negative correlation with systolic blood pressure.

In conclusion, we recommend that authors optimise every local, systemic, and environmental parameter which may have an effect on the results of such significant research.

## References

[1] Akçay B. İ. S., Kardeş E., Maçin S. (2016). Evaluation of subfoveal choroidal thickness in internal carotid artery stenosis. *Journal of Ophthalmology*.

[2] Tan K.-A., Gupta P., Agarwal A. (2016). State of science: choroidal thickness and systemic health. *Survey of Ophthalmology*.

[3] Nickla D. L., Wallman J. (2010). The multifunctional choroid. *Progress in Retinal and Eye Research*.

[4] Kee C. S., Marzani D., Wallman J. (2001). Differences in time course and visual requirements of ocular responses to lenses and diffusers. *Investigative Ophthalmology & Visual Science*.

